# Individualized network analysis: A novel approach to investigate tau PET using graph theory in the Alzheimer’s disease continuum

**DOI:** 10.3389/fnins.2023.1089134

**Published:** 2023-03-02

**Authors:** Hillary Protas, Valentina Ghisays, Dhruman D. Goradia, Robert Bauer, Vivek Devadas, Kewei Chen, Eric M. Reiman, Yi Su

**Affiliations:** ^1^Banner Alzheimer’s Institute, Phoenix, AZ, United States; ^2^Arizona Alzheimer’s Consortium, Phoenix, AZ, United States; ^3^Department of Neurology, The University of Arizona, Tucson, AZ, United States; ^4^Department of Psychiatry, The University of Arizona, Tucson, AZ, United States; ^5^Department of Neuroscience, School of Computing and Augmented Intelligence, Biostatistical Core, School of Mathematics and Statistics, College of Health Solutions, Arizona State University, Tempe, AZ, United States; ^6^Translational Genomics Research Institute, Phoenix, AZ, United States

**Keywords:** flortaucipir PET, graph theory, Alzheimer’s disease, tangle burden, ADNI

## Abstract

**Introduction:**

Tau PET imaging has emerged as an important tool to detect and monitor tangle burden in vivo in the study of Alzheimer’s disease (AD). Previous studies demonstrated the association of tau burden with cognitive decline in probable AD cohorts. This study introduces a novel approach to analyze tau PET data by constructing individualized tau network structure and deriving its graph theory-based measures. We hypothesize that the network- based measures are a measure of the total tau load and the stage through disease.

**Methods:**

Using tau PET data from the AD Neuroimaging Initiative from 369 participants, we determine the network measures, global efficiency, global strength, and limbic strength, and compare with two regional measures entorhinal and tau composite SUVR, in the ability to differentiate, cognitively unimpaired (CU), MCI and AD. We also investigate the correlation of these network and regional measures and a measure of memory performance, auditory verbal learning test for long-term recall memory (AVLT-LTM). Finally, we determine the stages based on global efficiency and limbic strength using conditional inference trees and compare with Braak staging.

**Results:**

We demonstrate that the derived network measures are able to differentiate three clinical stages of AD, CU, MCI, and AD. We also demonstrate that these network measures are strongly correlated with memory performance overall. Unlike regional tau measurements, the tau network measures were significantly associated with AVLT-LTM even in cognitively unimpaired individuals. Stages determined from global efficiency and limbic strength, visually resembled Braak staging.

**Discussion:**

The strong correlations with memory particularly in CU suggest the proposed technique may be used to characterize subtle early tau accumulation. Further investigation is ongoing to examine this technique in a longitudinal setting.

## 1. Introduction

Both Amyloid and tau begin building up long before clinical symptoms of Alzheimer’s disease (Jack et al., 2010, 2017b). Characterizing the level of tau pathology and its progression is of vital importance. Braak staging describes the spatial pattern of tau pathology and its spread from entorhinal and limbic to isocortical regions based on neuropathological studies (Braak and Braak, 1991, 1995). With the advent of tau PET tracers it is possible to measure the distribution of tangle burden *in vivo* and characterize its progression over time (Chien et al., 2013; Dani et al., 2016; Schwarz et al., 2018). Braak regions have been defined by FreeSurfer regions and conditional inference tree regression model (Scholl et al., 2016; Maass et al., 2017). Other *in vivo* Braak strategies have been adopted as well Schwarz et al. (2016, 2018). A meta-temporal region of interest which is defined as a collection of Braak III-IV regions has been proposed as a global index of overall tau burden (Jack et al., 2017a,b). While the spatial distribution of tau pathology generally follows the Braak stages, individual variability exists in where tau starts in the cortex and how it progresses (Braak et al., 2011; Franzmeier et al., 2020; Sanchez et al., 2021; Vogel et al., 2021). An alternative strategy to analyze tau PET data would still investigate tau spread throughout the brain in disease but allows for variability in the spatial pattern of tangle burden and does not necessarily follow Braak staging.

Graph-based or network approach can provide such an alternative and has been shown to allow detection of subtle changes of the brain antecede well known Alzheimer’s disease (AD) deficits (Morris et al., 2001; Albert, 2011; Arlt et al., 2013) without defined staging. This approach has been widely adopted to characterize the blood oxygen level dependent (BOLD) signal in resting state fMRI images for the investigation of brain function in health and disease (Wang et al., 2010, 2011; Braun et al., 2012; Hojjati et al., 2019). With this approach, a complex network can be constructed with nodes representing regional measures and edges describing relationship between nodes. Network-based measurements can then be used to identify modular structure in these networks (Wang et al., 2010, 2011; Farahani et al., 2019). In addition to its numerous applications in analyzing fMRI data, this approach has been reported for its use in analyzing T1w-MRI and PET data (He et al., 2007, 2008; Huang et al., 2010; Sepulcre et al., 2013, 2018; Tijms et al., 2013, 2018; Wang et al., 2016, 2020; Arnemann et al., 2018; Pereira et al., 2018; Veronese et al., 2019; Kim et al., 2021). Individualized network analyses are already available and very well established particularly in resting state fMRI and Diffusion Tensor Imaging(DTI) (Fornito et al., 2010; Braun et al., 2012; Hojjati et al., 2019). There are a few studies with T1-wMRI and FDG PET where individualized networks have been introduced (Tijms et al., 2012, 2013; Wang et al., 2016, 2020). The weights of both of these past individualized networks and group-based networks are defined by correlation, covariance, or other measures of similarity and association. The weights in new individualized tau networks introduced in this paper are instead characterized by difference in regional tau burden.

**FIGURE 1:**
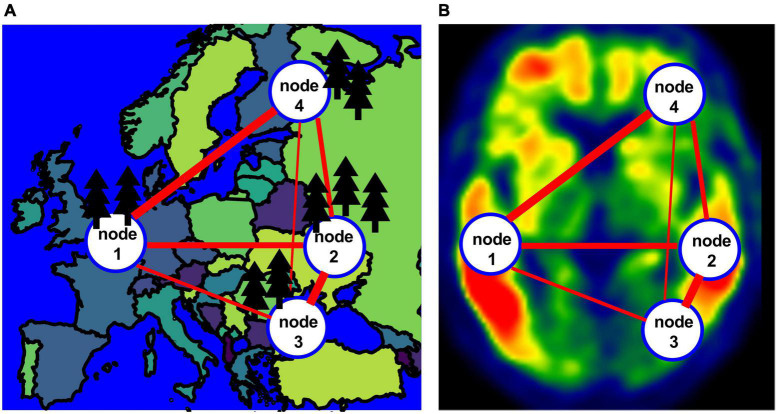
**(A)** An example of a population network with four nodes. **(B)** An example of the network for a tau image.

In this work, we propose a different approach to construct individual level tau PET networks drawing inspiration from and analogy to the application of graph-theoretical approach in the context of population genetics (Dyer and Nason, 2004; Dyer et al., 2010; Rodger et al., 2018; Savary et al., 2021a,b). The goal of analyzing tau PET data is to quantify tangle burden across different brain regions and make inference about how tangle burden would progress in a patient. Similarly in population genetics, researchers seek to characterize and quantify genetic variabilities in the overall population and describe genetic flow among different subpopulations using network -based approach for over 15 years (Dyer and Nason, 2004; Rodger et al., 2018; Savary et al., 2021a,b). In these research, a population graph or network, Figure 1A for an example, is constructed with each node representing a subpopulation and each edge connecting two nodes characterized by a distance measure representing the genetic difference between the two nodes (Excoffier et al., 1992; Dyer and Nason, 2004; Garroway et al., 2008; Dyer, 2009; Dyer et al., 2010; Manel and Holderegger, 2013; Shirk et al., 2018; Savary et al., 2021b). Therefore, similar to the application of graph-theoretical approach in population genetics research, we propose to construct individual level tau PET networks with each node representing different anatomical regions and the edge between the two nodes characterizing the difference in tau burden and then we derive graph-based metrics from the tau PET networks (Figure 1B). We examined the graph-based metrics and their ability to distinguish between cognitively unimpaired (CU), mild cognitive impairment (MCI) and AD participants in comparison to conventional regional analysis. In addition, we also examined the association of each individualized tau network measure with memory performance. We visually compare staging developed from the graph theory measures to Braak staging.

## 2. Materials and methods

### 2.1. Study participants

Data used in this study were obtained from participants in the Alzheimer’s Disease Neuroimaging Initiative (ADNI) database. Flortaucipir (FTP) PET scans were downloaded as of 11/29/2018 for 32 AD, 115 MCI and 223 CU older adults (Table 1). We included all participants with FTP scans from ADNI that also have a matching T1w MRI near the time of the FTP acquisitions (0.3 ± 0.6 years) regardless of the diagnostic status to allow us to examine tau pathology throughout the full AD continuum from biomarker negative cognitively normal participants to clinical AD. Matching T1w MRI and florbetapir (FBP) PET were also downloaded where available. This included participants with preclinical AD defined as those who were cognitively normal but had a positive AD biomarker, e.g., amyloid positive. Participants who did not fit into the typical AD/preclinical AD profile under the ATN framework (Jack et al., 2018) were also included. ADNI study was approved by the institutional review boards of the participating institutions. Signed informed written consent was obtained from each participant. For this project the most fully preprocessed PET images were downloaded from ADNI. The PET images underwent a procedure of between frame motion correction, averaging of the frames, reoriented to standard grid, and finally scanner harmonization filtering to 8 mm at University of Michigan. Details about the scan acquisition for MRI and PET are provided at the ADNI website.^1^ In addition to imaging data, demographic, clinical, and cognitive scores including auditory verbal learning test long term memory (AVLT-LTM) scores, Clinical Dementia Rating Scale-Sum of Boxes (CDR-SB), Mini Mental State examination (MMSE) score matching the imaging data were also downloaded from ADNI.

**TABLE 1:** Subject characteristics age, gender, APOE, education, cognitive measures as well as Aβ and tau positivity are included for the three groups (CU, MCI, and AD).

	CU (*n* = 222)	MCI (*n* = 115)	AD (*n* = 32)	*p*-value
Age	74.5 ± 7.4	76.9 ± 7.2	77.6 ± 9.4	0.003
Gender(M/F)	92/130	73/42	17/15	5e-4
APOE(NC/Car)	137/75	77/37	16/13	0.46
Education	16.7 ± 2.4	16.3 ± 2.7	15.4 ± 2.4	0.01
MMSE***	29.0 ± 1.3	27.8 ± 2.1	21.2 ± 4.8	4e-58
AVLT-LTM	10.0 ± 2.9	4.0 ± 4.0	0.9 ± 2.1	8e-51
AVLT-TL	46.6 ± 10.5	35.3 ± 10.4	22.2 ± 7.8	4e-35
CDR-SB	0.1 ± 0.3	1.2 ± 1.1	6.3 ± 3.1	9e-90
Aβ positivity(pos/neg) * (threshold: 1.17)	50/165	49/63	24/5	1e-10
Tau positivity(pos/neg)** (threshold:1.23)	104/118	80/35	27/5	2e-6

*Aβ positivity is defined by mean cortical region to/cerebellar region with a threshold for positivity of 1.17. **Tau positivity was defined based on the tau composite-SUVR greater than 1.23. ***CDR SB, clinical dementia rating sum of boxes; AVLT-TL, auditory verbal learning test total score, AVLT-LTM, auditory verbal learning test long term memory recall; MMSE, mini mental state examination.

### 2.2. Image analysis

The FTP image was co-registered to the nearest T1w MRI using SPM12 (Wellcome Trust Centre for Neuroimaging)^2^ and then SPM12 was used to warp MRI and the co-registered FTP image into Montreal Neurological Institute (MNI) template space. An in-house developed procedure was used to calculate the FTP standard uptake ratio (SUVR) values in template space for an entorhinal region (Braak et al., 2006; Johnson et al., 2016) and a tau composite-SUVR (Jack et al., 2017a,b). The entorhinal region is defined by the Mayo Clinic Adult Lifespan Template (MCALT) with a cerebral crus 1 reference region from Automated Anatomical Labeling (AAL) atlas (Tzourio-Mazoyer et al., 2002). The tau composite was computed as the median-uptake of voxels in entorhinal, amygdala, parahippocampal, fusiform, inferior temporal, and middle temporal regions normalized to cerebellar-crus following the previously described procedure (Jack et al., 2017a,b). This allowed us to determine Tau positivity, defined based on the tau composite-SUVR greater than 1.23 (Jack et al., 2017b). For those participants with a matching FBP scans, the FBP images were warped to the MNI template, amyloid burden was also determined using established methods and positivity is defined by having a mean cortical to cerebellum SUVR > 1.17 (Fleisher et al., 2011).

### 2.3. Individual-network based method

**FIGURE 2:**
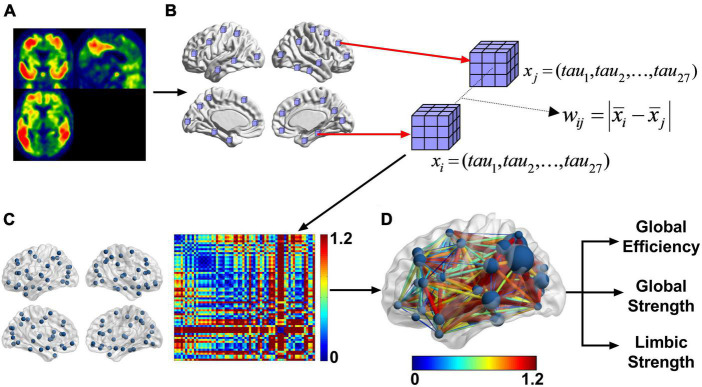
After preprocessing the PET images **(A)** into template space, we took 6 mm × 6 mm × 6 mm cubes centered at each of the AAL regions **(B)**. From that we calculated the difference between the mean tau SUVR of each cube. This resultant matrix is shown as a heatmap **(C)**. This is a weighted undirected network. The image in panel **(D)** is another representation of the weighted undirected matrix with the graph on the brain and the color representing the difference of regional tau SUVR. The size of the node is the strength. Each subject in this cross-sectional study had a graph for FTP and from that graph the global strength, global efficiency, and limbic strength can be calculated. Limbic strength is the strength over the bilateral parahippocampal and amygdala nodes.

A schematic of the method can be seen in Figure 2. The input to the pipeline is the warped tau SUVR image seen in 2A. A probabilistic gray matter mask (binarized with a threshold of 0.3) was used to define valid nodes of the network. For each of the 90 AAL regions (Tzourio-Mazoyer et al., 2002), a six-mm cube at its center (Xia et al., 2013; Yu et al., 2013) was subdivided into a 3 × 3 × 3 matrix with 2-mm isotropic elements as seen in Figure 2B. To be included in the network analysis, such cube must be a) independent of each other (no overlaps) and b) each of the 27 elements has a nonzero gray matter mask value. This left 64 regions (nodes) out of the 90 AAL regions (Supplementary Table 1).

To represent the tau network structure for each individual, an undirected network was constructed by calculating the weight (w) between each pair of nodes using a predefined weighting function. The weighting function is defined as the difference of the means of the two nodes:


(1) $$w_{i\,j}=\left|\bar{x_{i}}-\bar{x_{j}}\right|$$

Higher weight indicates a greater difference in tau deposition between the two nodes and vice versa. The inverse of the weight between two nodes defines the network distance. A larger tau interregional distance or weight corresponds with a shorter network distance. The shortest path length (*d*_*ij*_) is defined as minimum network distance between node i and j by either a direct or indirect route. For example, if *d_*ij*_* > *d*_*ik*_ + *d*_*kj*_ then *d*_*ij*_ is set to *d*_*ik*_ + *d_*kj*_*. In this case, the indirect path is the shorter path and replaces the direct path. The brain connectivity toolbox (BCT)^3^ (Rubinov and Sporns, 2010) was used to calculate efficiency, strength, and other measures of the network. Our method uses the weighted undirected network instead of binarized network to capitalize on the information carried by the weights.

Two types of network measures that are commonly used in network analysis are examined in this study and briefly summarized here.

#### 2.3.1. Network strength

The network strength of a node (nodal network strength) is defined as the sum of all the weights connected to a node:


(2) $$s_{i}=\sum_{j\in N}w_{i\,j}$$

*s_i_* Represents the strength of node i. Average nodal network strength of predefined sets of nodes can be calculated as a local or global measure. A local limbic strength is defined as the mean strength over four nodes (bilateral amygdala and parahippocampal gyrus) within the limbic network well-known to subject to tau pathology in AD (shown in Figure 2D). We use limbic strength as a representative network strength measure in the rest of the analysis to compare with conventional and other network-based tau measurements. A global strength defined as the mean nodal strength over all nodes is also examined as an overall strength measure of the network.

#### 2.3.2. Network efficiency

In a weighted network, global efficiency (*E^w^*) is a measure of the overall efficiency of information flow through nodes in the network (Eq. 3). It is the average of the inverse of the shortest path lengths. This is the new interregional distance matrix ($\tilde{w_{i\,j}}$) that includes both direct and indirect paths. $\tilde{w_{i\,j}}$ is inversely related to the shortest path length *d*_*ij*_ in the network, thus, a shorter path means higher efficiency. This measurement is very similar to network strength.


(3) $$E^{w}=\frac{1}{n}\,\sum_{i\in N}\frac{\sum_{j\in N,j\neq i}{\left(d_{i\,j}^{w}\right)}^{-1}}{n-1}=\frac{1}{n}\,\sum_{i\in N}\frac{\sum_{j\in N,j\neq i}\tilde{w_{i\,j}}}{n-1}$$

From these two types of network measures (strength and efficiency), in this study we focus on three measurements including global strength, limbic strength, and global efficiency.

### 2.4. Statistical analysis

To assess the utility and relevance of the three tau network measurements (global strength, limbic strength, and global efficiency), statistical analysis was performed to evaluate (1) the ability of these measurements in differentiating CU, MCI and AD groups and (2) the relationship between tau network measurements with cognitive measures. To evaluate the ability to differentiate CU, MCI and AD, Cohen’s *f* effect sizes, and confidence intervals were calculated for ANCOVA analysis for the three groups (CU, MCI, AD) with age and gender as a covariate using “effectsize” library in R. To characterize relationship of tau measures with AVLT-LTM scores (auditory verbal learning test for long-term recall memory), Spearman rank correlation were determined using R version 3.6.1 in CU and combined full groups. For the association analysis of Tau PET measures with memory in the CU group, we excluded participants who had a AVLT-LTM score of less than 5 (Ivnik et al., 1992). A Steiger test was used to compare the correlations for tau measures versus memory within groups using “cocor” library in R. Additional analysis was also performed to investigate the ability of using tau network measures for definition of pathological stages. The ctree function from the “partykit” library in R 3.6.1 was used to classify subjects using conditional inference tree based on the three tau network measures. After determining the stage, we averaged all tau PET images to create an average tau PET for each stage as well as averaging the tau strength for each node. We then created a surface display BrainNet with the nodes with average nodal strength as the size of the node.

## 3. Results

### 3.1. Participant characteristics

Demographic characteristics, MMSE, and AVLT-LTM scores are shown in Table 1. Age, education, and gender as well as cognitive and memory scores are significantly different between AD, MCI and CU groups (*p* < 0.05). The proportions of APOE carriers were not significantly different between the three groups (*p* = 0.46).

### 3.2. Tau network characteristics

**FIGURE 3:**
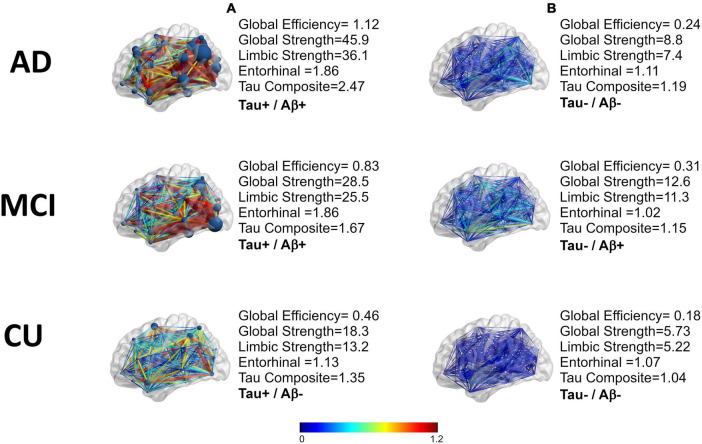
A representation of the tau networks for two subjects from each of the three clinical groups (AD,MCI, CU). The difference of the means of the weights are in color. The nodal strength can be seen as the size of the nodes. Tau positivity was defined based on the tau composite-SUVR greater than 1.23, **(A)** are tau positive and **(B)** are tau negative. Aβ positivity is defined by mean cortical region to/cerebellar region with a threshold for positivity of 1.17.

An example of the distance matrices of undirected networks derived from FTP images can be seen in Figure 2C. The tau networks are also displayed using BrainNet viewer (Xia et al., 2013) with the color representing the weights of the network and size of the nodes representing strength (Figures 2D, 3). Tau networks for a tau positive (left) and a tau negative (right) subject were randomly chosen from each of the clinical groups (i.e., CU, MCI, and AD) for illustration (Figure 3). Measurements for global efficiency, global strength, and limbic strength as well as the tau composite SUVR, entorhinal tau SUVR were also listed in Figure 3 for each selected subject. There is a general trend for AD patients than MCI and CU to have higher between-node distances. But it is not recommended to perform classification of patient clinical status based on this. And the network and regional tau measures are higher in tau positive than in tau negative participants. Subjects with higher tau regional measures tend to have higher tau network measures.

### 3.3. Individual network measurements—In AD, MCI, and CU groups

**FIGURE 4:**
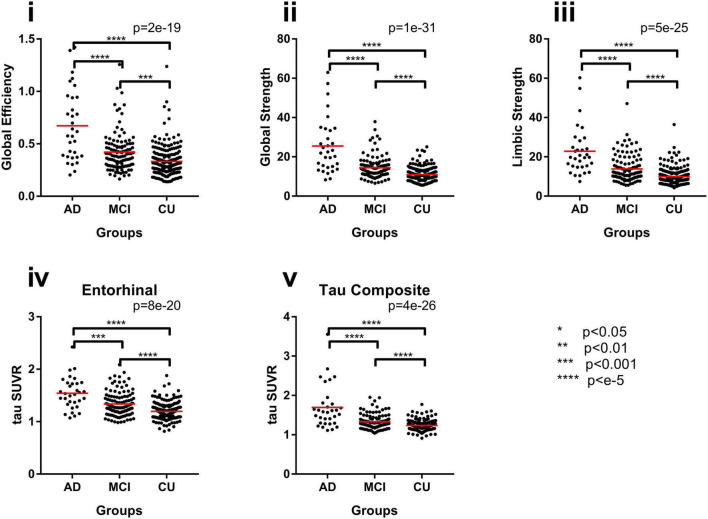
Group differences with network measures for the original FTP networks, **(i)** global efficiency, **(ii)** global strength, and **(iii)** limbic strength, and common regional measures, **(iv)** entorhinal SUVR and **(v)** tau composite SUVR between AD, MCI, and CU subjects. *p < 0.05, **p < 0.1, ***p < 0.002, ****p < e-5.

Group comparison for each tau network measurements including global efficiency, global strength, and limbic strength was performed controlling for age and gender (Figure 4). Tau composite SUVRs and entorhinal SUVRs were also compared among the three groups (Figure 4). All tau network measures showed significant difference among the 3 groups (*p* < 2.0e-19, ANCOVA), as well as the pairwise separation (*p* < 0.001). Of note, all network measures (i.e., global efficiency, global strength) increase from CU to AD. The tau SUVR measures also showed similar group separation (*p* < 8.0e-20, ANCOVA) and pairwise separation (*p* < 0.001). In Table 2, we see the Cohen’s *f* based on ANCOVA analysis for the 3 group with age and gender as the covariate. All network and regional measures show a large effect (Cohen’s *f* > 0.4) for separation among AD, MCI and CU. Tau global strength demonstrated largest Cohen’s *f* (0.69, Table 2), followed by tau composite SUVR (0.62). Table 3 shows the effect size for the pairwise analyses CU versus MCI and AD versus MCI. The tau measures with largest effect size in analysis between MCI and CU is tau global strength and entorhinal SUVR (Cohen’s *f* = 0.34) followed by tau limbic strength (Cohen’s *f* = 0.33). The tau measures with largest effect size in analysis between MCI and AD is tau global strength (Cohen’s *f* = 0.57) followed by) and tau composite SUVR (Cohen’s *f* = 0.51). Network (global strength and limbic strength) and regional measures have clinically relevant effect sizes for both pairwise group comparisons (AD vs. MCI groups and MCI vs. CU groups). Global efficiency has lower effect sizes in CU/MCI pairwise assessment. As expected, the best regional measurement for effect size analyses of CU and MCI is entorhinal and the best regional SUVR measurement for effect size analyses of MCI vs. AD is tau composite SUVR.

**TABLE 2:** Effect sizes, Cohen’s *f*, from either FTP PET network measures or regional measure volumes for the for ancova of the three groups (AD, MCI, CU).

	AD/MCI/CU
Tau global efficiency	0.52 (0.40, 0.624)*
Tau global strength	0.69 (0.575, 0.80)
Tau limbic strength	0.60 (0.49, 0.71)
Tau composite SUVR	0.62 (0.50, 0.73)
Tau entorhinal SUVR	0.52 (0.41, 0.63)

Cohen’s *f*, 95% confidence intervals are shown and covariates included in the analysis were age and gender. *Cohen’s *f* (95% confidence intervals).

**TABLE 3:** Effect sizes, Cohen’s *f*, from either FTP PET network measures or regional measure volumes for the for pair wise analysis of the three groups (AD, MCI, CU).

	AD/CU	AD/MCI	MCI/CU
Tau global efficiency	0.62 (0.49, 0.76)*	0.47(0.29, 0.64)	0.22 (0.11, 0.33)
Tau global strength	0.82 (0.67, 0.96)	0.57 (0.39, 0.75)	0.34(0.23, 0.45)
Tau limbic strength	0.74 (0.60, 0.88)	0.46 (0.29, 0.64)	0.33 (0.22, 0.44)
Tau composite SUVR	0.70 (0.56, 0.84)	0.51 (0.33, 0.68)	0.31 (0.20, 0.42)
Tau entorhinal SUVR	0.64 (0.50, 0.77)	0.34 (0.17, 0.51)	0.34 (0.23, 0.45)

Cohen’s *f*, 95% confidence intervals are shown and covariates included in the analysis were age and gender. *Cohen’s *f* (95% confidence intervals).

### 3.4. Relationship of tau PET (network and regional) to memory

**FIGURE 5:**
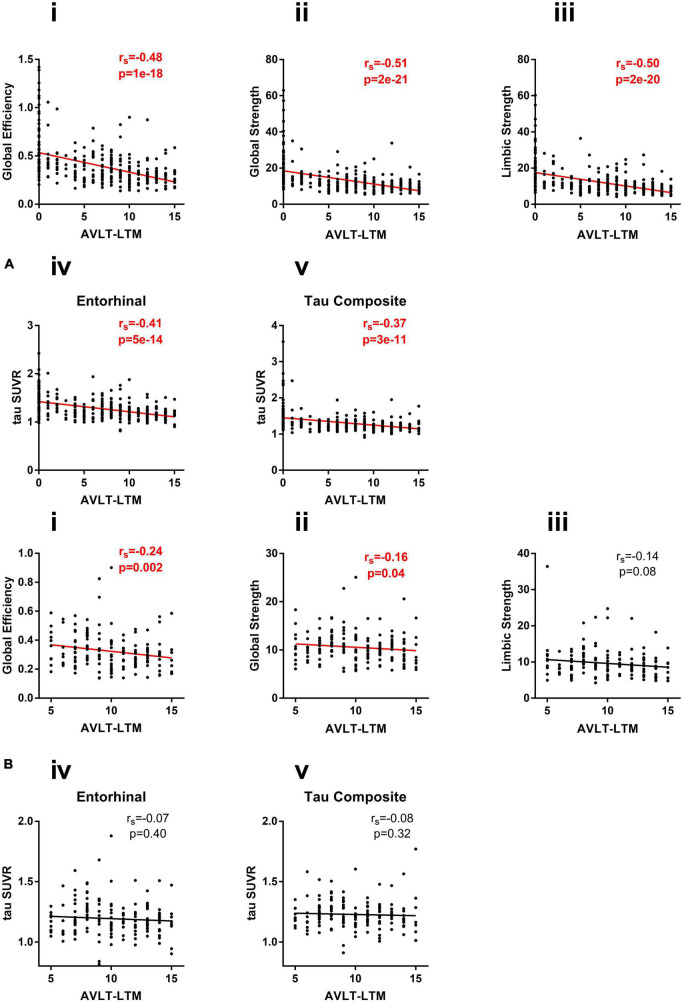
**(A)** Correlation of network measures (i) global efficiency, (ii) global strength, and (iii) limbic strength, and regional measures (iv) entorhinal SUVR, and (v) tau composite SUVR with AVLT-LTM in all subjects (CU/MCI/AD). **(B)** Correlation of network measures (i) global efficiency, (ii) global strength, and (iii) limbic strength and regional measures (iv) entorhinal SUVR, and (v) tau composite SUVR with AVLT-LTM in CU subjects.

The association of tau measures with AVLT-LTM in full group as well as within CU subjects separately was shown in Figure 5. In the full group where AVLT-LTM has values covering the full range, all tau measures were significantly associated with AVLT-LTM (Figure 5A). The correlation between global efficiency and AVLT-LTM is significantly stronger than the correlation between AVLT-LTM and tau composite (*p* < 0.04). The correlations between both global strength and limbic strength and AVLT-LTM are also significantly stronger than the correlation between AVLT-LTM and regional measurements (*p* < 0.04). In CU, only two tau network measures were significantly associated with AVLT-LTM, (tau global efficiency: *r*_s_ = −0.24, *p* = 0.002, tau global strength: *r*_s_ = −0.16, *p* = 0.04, Figure 5B), other tau measures did not show a significant association. The results were similar when restricted to amyloid positive participants only. All tau measures were significantly associated with AVLT-LTM (*p* < 0.05) in amyloid positive participants across the clinical spectrum. In amyloid positive CU participants, only limbic strength is correlated with AVLT-LTM (*r*_s_ = −0.37, *p* = 0.03), global efficiency and strength as well as tau SUVR measures was not significantly associated with AVLT-LTM.

### 3.5. Staging with tau PET measures

**FIGURE 6:**
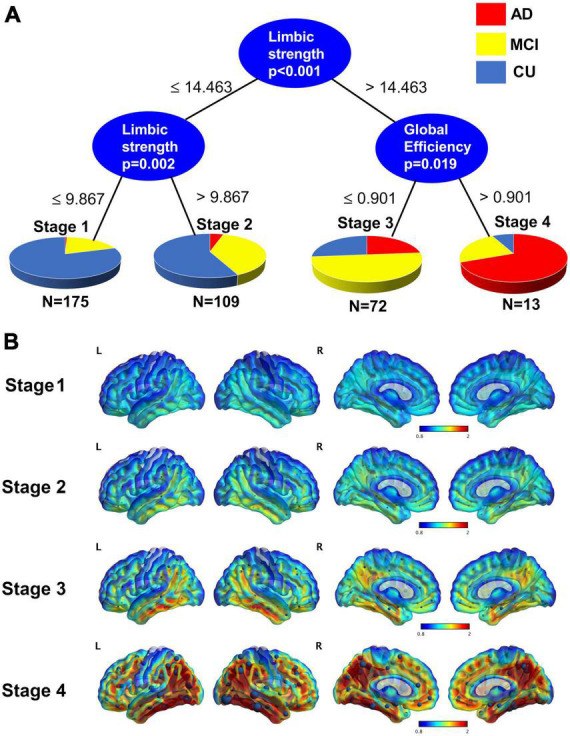
**(A)** Conditional inference trees model for staging using limbic strength and global efficiency. A threshold of 14.463 for limbic strength separates those participants who are in stage 1 and 2 from those who are stage 3 and 4. Those lower than the 14.463 are further divided into stage 1 and stage 2 by the threshold 9.867. Those greater 14.463 are divided into stage 3 and stage 4 with threshold of global efficiency = 0.901. **(B)** Average tau surface maps for each stage. On top of the surface display, the average strength from each node are displayed. Stage 4 is particularly like the Braak V-VI.

We show the staging based on using global efficiency, and limbic strength as seen in Figure 6A. There are two thresholds for limbic strength, first a threshold = 14.46 to divide participants into stage 1–2 and 3–4 and then a threshold = 9.867 to divide stage 1-2 into stage 1 and stage 2. A threshold of global efficiency = 0.901 was found to divide participants into stage 3 and stage 4. The average tau PET image for each of the 4 stages defined based on tau network measures was illustrated in Figure 6B. The spatial pattern of tau burden across the 4 stages resembles the pattern described by Braak staging with tau accumulation starting from the medial temporal lobe and spreading to the isocortical regions. It appears that on average in the early stages there might be some tau starting not only in medial temporal lobe but also precuneus.

## 4. Discussion

In this paper we introduce individualized tau network measurements using graph theory that allows the assessment of tangle burden across the entire brain. Global strength, global efficiency, and limbic strength in the tau networks are higher in AD subjects (AD > MCI > CU). Global strength, global efficiency and limbic strength had similar effect sizes compared to traditional regional SUVR measures of tau burden. The network measures and regional measures showed similar discrimination between AD and CU. As an example of the discriminative ability between AD and CU of the tau measures, limbic strength demonstrated an AUC (95% confidence interval) = 0.90 [0.83, 0.97], specificity = 0.88, sensitivity = 0.83. In general, both tau network measures and the regional SUVR measures correlated with memory score, while only the network measures (global efficiency, global strength were significantly correlated with memory in the CU group). These findings suggest our proposed tau network analysis approach can be a useful technique in providing an overall assessment of tau burden with strong statistical power in differentiating clinical AD stages and predicting cognitive performance. Further investigation with larger cohorts may be able to determine whether network-based measurements had superior statistical power for group separation. We also demonstrated our approach to be more sensitive to detecting the relationship between tangle burden and cognitive decline prior to clinical symptoms. Visually, the stages determined based on global efficiency and limbic strength also resemble the general pattern of tau progression as defined based on Braak staging. Fifty three percent of the CU in stage 3 and 4 are Aβ positive, 47% of the CU in stage 3 and 4 are APOE4 carriers. 90% of the CU in stage 3 and stage 4 are tau positive. It should be noted that the gold standard for Braak staging should be based on neuropathological assessment while tau PET can partially replicate that although it should be interpreted with caution (Lowe et al., 2020; Soleimani-Meigooni et al., 2020). Numerous studies have shown deviations from typical Braak staging based spatial patterns and individual variability exists in where tau starts in the cortex and how it progresses (Braak et al., 2011; Franzmeier et al., 2020; Sanchez et al., 2021; Vogel et al., 2021). The goal of performing staging based on network measure in this study is mainly to demonstrate its similarity with the Braak based staging system. Further study is warranted to examine the validity and performance of this staging system using both cross-sectional and longitudinal data.

In the association analysis between tau measures and AVLT-LTM, excluding amyloid negative participants did not substantially change the observed association. All tau measures were associated with AVLT-LTM across the clinical spectrum and only certain tau network measures can detect an association in the CU group. While focusing on amyloid positive participants allows us to investigate the spectrum of patients fitting the typical pathological profile of AD it also results in reduced sample size and preventing us from examine the very early phase of the disease. The subtle difference of which network measure were associated with AVLT-LTM in the CU group depending on the amyloid status may be a results of sample size differences or underlying differences in tau distribution pattern that require further investigation.

We primarily examined AVLT-LTM which is more sensitive to earlier cognitive changes. AVLT-LTM is a sensitive measure to detect memory decline including at early stages when participants are still considered cognitively normal (Caselli et al., 2009). We also examined correlation with Clinical Dementia Rating Scale-Sum of Boxes (CDR-SB), Mini Mental State examination (MMSE), AVLT-total, and AVLT-STM. The results were consistent with our findings with AVLT-LTM. All cognitive measures were significantly correlated with conventional tau SUVR measures as well as network based measures, while the relationship tend to be stronger for network measures. Also, in CU participants, only network-based measures were found to be significantly correlated with cognitive measurements.

In our proposed technique to derive individualized tau networks, the nodes were defined based on average gray matter maps in MNI template space with each node centered within each individual AAL region, and each node is represented by a cube in the imaging space. We also investigated the influence of tau PET analysis pipelines and choice of nodes for the construction of the individualized tau networks. These results were summarized in the Supplementary material. We found our approach to be generally robust and not sensitive to the detailed variation in the implementation of the network model such as the size of the cube, the number of nodes, and template space vs. individual space analysis. These findings support the generalized application of our proposed approach to different tau analysis pipelines without losing its power to detect tau changes and its association with cognition. We did not compare in this manuscript the individualized network with a group-based network like tau covariance since the group based networks and individual based networks serve different purposes and the goal of this manuscript was to demonstrate the utility of our individual based networks which allows the assessment of tau burden and its spatial distribution at individual level. Group based networks only examine the spatial pattern of tau distribution. A comparison of the two approaches is beyond the scope of our current study although would be interesting for future study.

In network analysis pruning of edges or weights is a commonly performed task. To examine the sensitivity of our tau network analysis approach to such pruning, we included two different implementation of individualized tau network with one using 86 FreeSurfer defined nodes including both cortical and subcortical regions and another one focusing on the cortical regions only given the off-target binding in subcortical regions (Baker et al., 2019). The results are largely similar in that the network metrics’ ability to differentiate clinical groups and correlating with memory scores are not affected by the two implementations.

Although tau PET tracers are designed to target tau fibril tangles, tau PET images nevertheless carry structural information due to the difference in non-specific tracer retention among different tissue types, e.g., gray vs. white matter. To better understand whether the network measures derived from tau PET images are primarily driven by specific tau binding rather than non-specific signal which would resemble more of structural MR data, we applied the same approach to construct individual level gray matter and white matter networks based on tissue density map derived from T1w-MRI, and subsequently evaluated these T1w-MRI derived network measures in their ability to differentiate AD diagnostic groups and their association with cognition (Supplementary Tables 5–7). It was determined that the T1w-MRI derived network measures had far less power in differentiate the clinical groups (Supplementary Table 5). We also found that the T1w-MRI derived network measures had weaker or no association with cognition and sometimes in the opposite direction (Supplementary Tables 6, 7). These observations suggest that our proposed tau network measures are driven more by the specific tau-binding related signals which further strengthens their utility in assessing tau pathologies.

In summary, we proposed and evaluated a novel approach to construct individualized tau network and compute graph-theoretical measures of the network. We found these measures to differentiate clinical stages and have stronger associations with cognition compared to regional measures, particularly in CU. In addition, this method can be applied to other tauopathies and subtypes of AD that show differing patterns of tau deposition. Additional investigation is needed to relate these tau network measures with other AD related changes such as amyloid and neurodegeneration as well as determine its power in track longitudinal changes of tangle burden. This method can be further extended to a longitudinal network to measure longitudinal tau spread. For future work we will also examine the ability of using network measures to determine tau positivity. Further investigation is also warranted to determine whether these subtle tau changes identified by network analysis can be detected in other tauopathies.

## Data availability statement

The original contributions presented in this study are included in the article/Supplementary material, further inquiries can be directed to the corresponding author.

## Ethics statement

The studies involving human participants were reviewed and approved by the Alzheimer’s Disease Neuroimaging Initiative. The patients/participants provided their written informed consent to participate in this study.

## Author contributions

HP: methodology, conceptualization, investigation and analysis and writing. VG: investigation and writing original draft. DG, KC, and ER: investigation and reviewing and editing. RB and VD: data management. YS: methodology, conceptualization, investigation, supervision, and writing—review and editing. All authors contributed to the article and approved the submitted version.

## Footnotes

1
www.adni-info.org

2
http://www.fil.ion.ucl.ac.uk/spm/

3
http://www.brain-connectivity-toolbox.net/
