# Supplementary material for: Individualized network analysis: A novel approach to investigate tau PET using graph theory in the Alzheimer’s disease continuum
Source: Front Neurosci. 2023 Mar 2;17:1089134. doi: 10.3389/fnins.2023.1089134 (PMC10017746; doi:10.3389/fnins.2023.1089134)
Supplement: Supplementary file 1 [file Data_Sheet_1.pdf]

Supplementary Material

1 Investigating the impact of technical variation in the implementation of individualized tau PET network

The primary implementation of individualized tau PET network was based on spatially normalized tau PET SUVR images divided into 64 nodes (Supp. Table 1) defined by AAL atlas with six-mm cube for each node. To examine the sensitivity of our individualized tau PET network analysis approach to the technical details of tau PET analysis and network construction, we examined five alternative implementations of our general approach: 1) 64 nodes from AAL atlas (see supplemental table 1 for the specific regions) 2) 90 nodes from AAL atlas all but the cerebellum, 3) 136 regions from the AAL3 atlas which include the AAL 90 regions and also including orbitofrontal, 30 thalamus, 6 anterior cingulate cortex subregions , nucleus accumbens, VTA , Substantia nigra , and Red nucleus (Rolls et al., 2020), and 4) 86 nodes based on FreeSurfer (FS) defined regions in individual space – 68 cortical regions plus thalamus, putamen, pallidium, caudate, amygdala, hippocampus, inferior lateral ventricle, and lateral ventricle (both left and right) 5) on the 68 nodes based on FS defined cortical regions in individual space- no subcortical regions were included (Supp. Fig. 1). Both FS SUVR were downloaded from Berkeley (FS 7.1.1) and had inferior cerebellum reference region. The weights are the absolute value of the difference of the mean tau SUVR of each region. For the AAL3 regions we did try most of the AAL3 regions except LC and Raphe. Network measures were calculated for each variation of the network definition, and their ability to differentiate clinical groups and correlate with cognition were assessed. The results were summarized in Supp. Tables 2-4. Similar results were found with these alternative implementations. The network measures were able to differentiate clinical groups and correlate with cognitive measure. For CU, we found that global efficiency had the highest correlation of the memory for each method, but only global efficiency derived from the cubes had significantly higher correlation than entorhinal SUVR using a Steiger test. These networks were very comparable, with the derivation with the cubes centered around 64 AAL regions possibly a bit better in CU with respect to correlation with AVLT-LTM. But more work is necessary to determine the best weighted network.

Supplementary Table 1: 64 nodes included in the network.

Precentral_L	Supp_Motor_Area_R	Amygdala_R	Fusiform_R	Putamen_L
Frontal_Sup_L	Frontal_Med_Orb_L	Calcarine_L	Postcentral_R	Heschl_L
Frontal_Sup_R	Frontal_Med_Orb_R	Calcarine_R	Parietal_Sup_L	Heschl_R
Frontal_Mid_L	Rectus_L	Cuneus_L	Parietal_Sup_R	Temporal_Sup_L
Frontal_Mid_R	Rectus_R	Lingual_L	Parietal_Inf_L	Temporal_Sup_R
Frontal_Mid_Orb_R	Insula_L	Lingual_R	Parietal_Inf_R	Temporal_Pole_Sup_L
Frontal_Inf_Oper_L	Insula_R	Occipital_Sup_L	SupraMarginal_L	Temporal_Pole_Sup_R
Frontal_Inf_Oper_R	Cingulum_Ant_L	Occipital_Sup_R	SupraMarginal_R	Temporal_Mid_L
Frontal_Inf_Tri_L	Cingulum_Ant_R	Occipital_Mid_L	Angular_L	Temporal_Mid_R
Frontal_Inf_Tri_R	Cingulum_Mid_L	Occipital_Mid_R	Angular_R	Temporal_Pole_Mid_L
Frontal_Inf_Orb_L	ParaHippocampal_L	Occipital_Inf_L	Precuneus_L	Temporal_Pole_Mid_R
Frontal_Inf_Orb_R	ParaHippocampal_R	Occipital_Inf_R	Precuneus_R	Temporal_Inf_R
Supp_Motor_Area_L	Amygdala_L	Fusiform_L	Caudate_L	

Supplemental Figure 1: Investigations into a few alternative methods to determining the network as well. We wanted to see whether we were missing the signal with such small cubes by using the mean of the full region to calculate the weight. The nodes were calculated from the AAL atlas 1) the same 64 regions used for the cubes 2) 90 regions all but the cerebellum and 136 regions from the AAL3 atlas(Rolls et al., 2020) (the smallest regions were not included in the derivation of the network). In addition to template-based derivation of the network we also looked into whether FS based individual space regions could be used. The Berkeley tau FS measurements (non PVC) were used to determine a network from 86 regions cortical and subcortical regions. We also show only the 68 cortical regions as well. Here we show the distribution of the different number of the nodes. The nodes location are determined by the center of gravity from FSL.

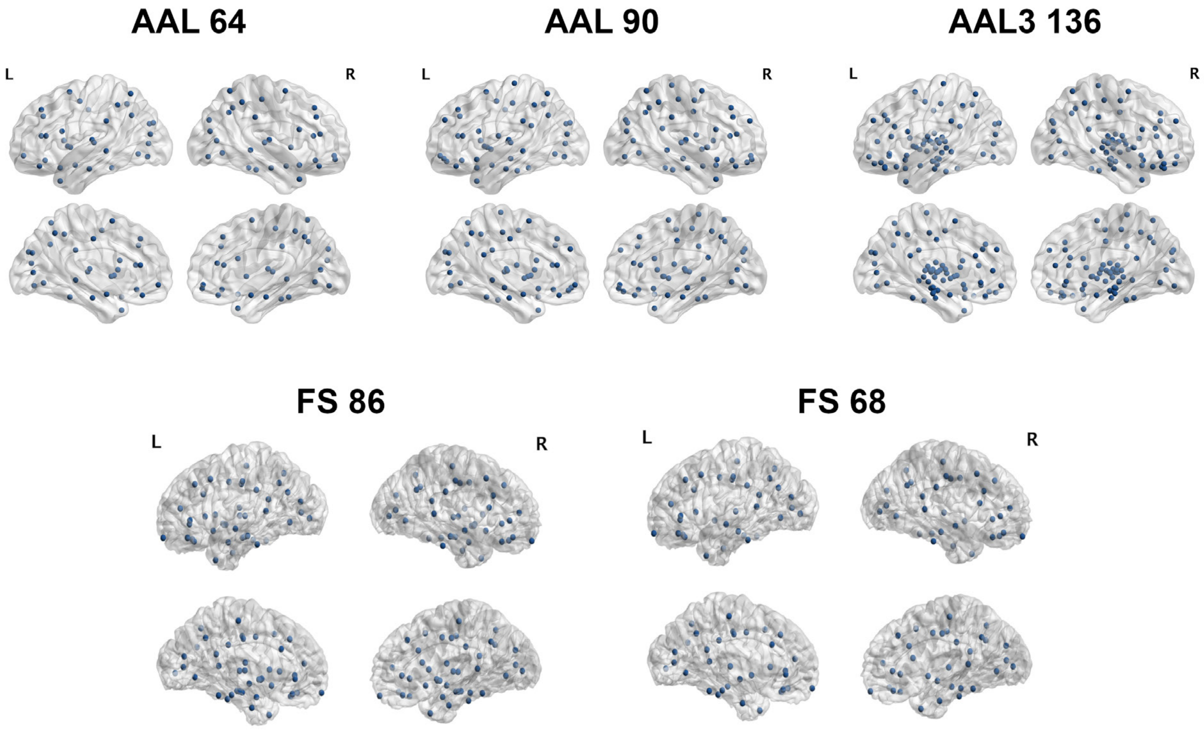

Supplemental Table 2: We compare the effect sizes – Cohen's f, 95% confidence intervals - covariates-age and gender for all 5 methods. 64 cubes represents the method eventually adopted for the paper. For the methods based with FS, we also looked at the entorhinal strength. FS86 and FS68 are only methods with the entorhinal node. For FS68, limbic strength is only parahippocampal strength since there is no amygdala.

	**Global Strength**	**Global Efficiency**	**Limbic strength**	**Entorhinal strength**
**64 cubes**	0.69(0.575, 0.804)	0.52(0.404, 0.624)	0.60(0.486, 0.71)	
**AAL 64**	0.65(0.539, 0.765)	0.50(0.386, 0.605)	0.54(0.429, 0.649)	
**AAL 90**	0.60(0.483, 0.707)	0.38(0.265, 0.478)	0.55(0.439, 0.66)	
**AAL 136**	0.42(0.311, 0.526)	0.37(0.255, 0.468)	0.55(0.436, 0.657)	
**FS 86**	0.66(0.541, 0.768)	0.52(0.411, 0.631)	0.64(0.525, 0.751)	0.61(0.493, 0.717)
**FS 68**	0.68(0.567, 0.795)	0.61(0.498, 0.723)	0.64(0.52, 0.746)	0.60(0.485, 0.709)

* Cohen's f (95% confidence intervals)

Supplemental Table 3: Correlation with memory (AVLT-LTM) for all alternative derivations of the network (64 cube) proposed in this paper over all subjects. Spearman correlation is given for each fit

		Global Strength	Global Efficiency	Limbic strength	Entorhinal strength
**64 cubes**	r_s	-0.508	-0.476	-0.497	
	pvalue	1.53E-21	9.95E-19	1.60E-20	
**AAL 64**	r_s	-0.48	-0.493	-0.474	
	pvalue	3.88E-19	3.12E-20	1.29E-18	
**AAL 90**	r_s	-0.458	-0.404	-0.486	
	pvalue	2.48E-17	1.81E-13	1.22E-19	
**AAL3 136**	r_s	-0.379	-0.407	-0.486	
	pvalue	6.62E-12	1.12E-13	1.24E-19	
**FS 86**	r_s	-0.518	-0.488	-0.509	-0.482
	pvalue	1.68E-22	9.37E-20	1.20E-21	2.92E-19
**FS 68**	r_s	-0.471	-0.473	-0.439	-0.457
	pvalue	2.50E-18	1.35E-18	6.32E-16	2.92E-17

Supplemental Table 4: Correlation with memory (AVLT-LTM) for all alternative derivations of the network (64 cube) proposed in this paper over CU subjects. Spearman correlation is given for each fit

		**Global Strength**	**Global Efficiency**	**Limbic strength**	**Entorhinal strength**
**64 cubes**	r_s	-0.157	-0.237	-0.137	
	pvalue	4.39E-02	2.21E-03	7.98E-02	
**AAL 64**	r_s	-0.129	-0.201	-0.081	
	pvalue	9.94E-02	9.53E-03	3.03E-01	
**AAL 90**	r_s	-0.168	-0.18	-0.106	
	pvalue	3.07E-02	2.07E-02	1.74E-01	
**AAL 136**	r_s	-0.099	-0.157	-0.094	
	pvalue	2.04E-01	4.41E-02	2.30E-01	
**FS 86**	r_s	-0.169	-0.182	-0.123	-0.126
	pvalue	2.99E-02	1.96E-02	1.15E-01	1.06E-01
**FS 68**	r_s	-0.102	-0.078	-0.14	-0.094
	pvalue	1.91E-01	3.17E-01	7.37E-02	2.29E-01

2 Investigating the signal contribution to individualized tau PET network measures

To confirm that the tau PET network measures were primarily driven by tau specific signal rather than nonspecific tracer retention differences across brain tissue type and participants we applied the same approach to construct individual level gray matter and white matter networks based on tissue density map derived from T1 MRI, and subsequently evaluated these T1-MRI derived network measures in their ability to differentiate AD diagnostic groups and their association with cognition. We segmented the MRI in to gray and white matter with spm12. The warp from the segmentation was applied to both the gray and white matters. We tried the same variety of template-based methods for tau PET, but found the best seemed to be the full 90 AAL regions for gray matter, and full 64 AAL regions for white matter. The weights for the gray and white networks were the absolute difference between each region. The results are summarized in Supp. Table 5-7.

Supplemental Table 5: Compare effect sizes of network measures from either FTP PET, gray matter volume or white matter volumes– Cohen's f for the three groups (AD, MCI, CU), 95% confidence intervals - covariates-age and gender. The gray matter volume and white matter were segmented with SPM12 and then warped to template space. And then the same variety template-based network were applied to both gray and white matter. The networks with the best group separation and correlation with memory was chosen for both gray matter (90 AAL) and white matter (64 AAL regions) and shown below only.

	**Global Strength**	**Global Efficiency**	**Limbic strength**
Tau	0.69(0.575, 0.804)	0.52(0.404, 0.624)	0.60(0.486, 0.71)
Gray matter	0.11 (0, 0.204)	0.22 (0.101, 0.314)	0.28 (0.163, 0.376)
White matter	0.18 (0.0622, 0.277)	0.14 (0.00154, 0.24)	0.17 (0.0546, 0.271)

* Cohen's f (95% confidence intervals)

Supplemental table 6: Correlation with memory (AVLT LTM) between network measures derived from tau PET, gray matter volumes , or white matter volumes for all subjects. Spearman correlation is given for each fit.

		**Global Strength**	**Global Efficiency**	**Limbic strength**
Tau	r_s	-0.508	-0.476	-0.497
	pvalue	1.53E-21	9.95E-19	1.60E-20
Gray matter	r_s	0.214	0.266	0.243
	pvalue	1.40E-4	1.82E-6	1.43E-5
White matter	r_s	-0.186	-0.259	-0.120
	pvalue	9.4E-4	3.42E-6	3.48E-2

Supplemental table 7: Correlation with memory (AVLT LTM) between network measures derived from tau PET, gray matter volumes , or white matter volumes for CU subjects. Spearman correlation is given for each fit.

		**Global Strength**	**Global Efficiency**	**Limbic strength**
Tau	r_s	-0.157	-0.237	-0.137
	pvalue	4.39E-02	2.21E-03	7.98E-02
Gray matter	r_s	0.107	0.149	0.043
	pvalue	0.162	0.05	0.57
White matter	r_s	-0.10	-0.18	-0.04
	pvalue	0.18	0.02	0.60

Reference

Rolls, E.T., Huang, C.C., Lin, C.P., Feng, J., and Joliot, M. (2020). Automated anatomical labelling atlas 3. *Neuroimage* 206**,** 116189. doi: 10.1016/j.neuroimage.2019.116189.
